# Placenta Peptide Can Protect Mitochondrial Dysfunction through Inhibiting ROS and TNF-α Generation, by Maintaining Mitochondrial Dynamic Network and by Increasing IL-6 Level during Chronic Fatigue

**DOI:** 10.3389/fphar.2016.00328

**Published:** 2016-09-27

**Authors:** Rekik A. Muluye, Yuhong Bian, Li Wang, Paulos N. Alemu, Huantian Cui, Xiaofei Peng, Shanshan Li

**Affiliations:** ^1^Tianjin University of Traditional Chinese MedicineTianjin, China; ^2^Ethiopian Public Health Institute Addis Ababa, Ethiopia; ^3^Tianjin Second People’s HospitalTianjin, China

**Keywords:** chronic fatigue, mitochondria, placenta peptide, ROS, cytokines

## Abstract

**Background:** Level of fatigue is related to the metabolic energy available to tissues and cells, mainly through mitochondrial respiration, as well fatigue is the most common symptom of poorly functioning mitochondria. Hence, dysfunction of these organelles may be the cause of the fatigue seen in Chronic fatigue (CF). Placenta has been used for treatment of fatigue and various disease, moreover peptides has known protect mitochondrial viability, and alleviate fatigue. These properties of placenta and peptides may link with its effect on mitochondria; therefore, it is highly important to investigate the effectiveness of placenta peptide on fatigue and mitochondrial dysfunction.

**Methods:** After administration of sheep placenta peptide (SPP) for 1 month, mice’s were forced to swim till exhaustion for 90 min to induce chronic fatigue. Electron microscopic examination of skeletal muscle mitochondrial structure, tissue Malondialdehyde (MDA), mitochondrial SOD and serum inflammatory cytokines level were investigated in order to determine the potential effect of SPP on mitochondria during CF. Rat skeletal muscle (L6 cell) were also treated with different concentration of SPP to determine the effect of SPP on cell viability using Thiazoyl blue tetrazolium assay.

**Results:** Our finding revealed that forced swimming induced fatigue model can cause mitochondrial damage through Reactive oxygen species (ROS) mediated lipid peroxidation and Tumor Necrosis factor alpha (TNF-α) elevation. Whereas SPP protected fatigue induced mitochondrial dysfunction through preventing ROS and TNF-α generation, by maintaining mitochondrial dynamic network and by increasing serum IL-6 level.

**Conclusion:** SPP can protect damage in mitochondrial components which will allow proper functioning of mitochondria that will in turn inhibit progression of chronic fatigue. Therefore, SPP may represent a novel therapeutic advantage for preventing mitochondrial dysfunction in patients with chronic fatigue.

## Introduction

Chronic fatigue is a clinically defined condition characterized by persistent, severe, disabling fatigue lasting more than 6 months that is not reversed by sleep or rest (Fukuda et al., 1994; Norheim et al., 2011).

It is also an important secondary condition in many clinical diagnoses and occurs naturally during aging. Fatigue is a complex phenomenon determined by several factors, including psychological health but at the biochemical level fatigue is related to the metabolic energy available to tissues and cells, mainly through mitochondrial electron transport (Chazotte, 2001; Finsterer, 2012). Fatigue is the most common symptom of poorly functioning mitochondria; the classic symptoms of persistent and debilitating fatigue, chronic muscle weakness, and myalgia are consistent with mitochondrial dysfunction in other diseases of known mitochondrial etiology (Chazotte, 2001; Nicolson and Ellithorpe, 2006). Most fatigue patients report mental concentration impairment and cognitive deficits, which are also seen in mitochondrial dysfunctions (Nicolson and Ellithorpe, 2006). Thus the integrity of mitochondrial is critical to cell function and energy metabolism.

Even though fatigue decreases the quality of life in people there are only few pharmacological drugs or therapies available for the treatment of fatigue (Fukuda et al., 1994; Kim et al., 2005; Reeves et al., 2007; Uthayathas et al., 2007). It has been showed that, placenta has protective effect on chronic fatigue, and many other diseases (Biswas et al., 2001; Kong et al., 2008; Rozanova et al., 2012). Moreover, peptides protect mitochondrial viability, and minimize further ROS generation (You et al., 2011). These properties of placenta and peptides may link with its effect on mitochondria; therefore it is highly important to investigate the effectiveness of this drug on fatigue and mitochondrial dysfunction.

The aim of this study was, to evaluate the potential effect of SPP on mitochondrial dysfunction in forced swimming induced fatigue model as well, to distinguish the underlying mechanism by assessing mitochondrial structure and functioning through ultra structural analysis of skeletal muscle mitochondria, ROS and pro-inflammatory cytokines determination and investigating SPP effect on cell viability.

## Materials and Methods

### Reagent and Chemicals

Coenzyme Q10 powder from Sangon (Sangon biotech, Shang Hai, China). Essential Medium Alpha (α MEM), Fetal bovine serum (FBS) and Penicillin/streptomycin (P/S) from Hyclone (Thermo Scientific Hyclone, US), Non-essential amino acid (NEAA) from Gibco (life technologies, Grand Island, NY, USA), ELISA kit for IL- 6 and TNF-α determination from xiao deng (xiao deng, china), mtSOD and tissue MDA determination kit from Cominbio (Suzhou Comin Biotechnology, Zhang Su, China) were used in the study. All medium, chemical, and kits used compiled with the required standard and were of analytical grade.

### Sheep Placenta Peptide Preparation

SPP was prepared and supplied by Tianjin jiani (Tianjin jiani Biological Science and Technology Co., LTD, Tianjin, China.). SPP was extracted by enzyme hydrolysis method, briefly fresh sheep placenta washed, cut into small pieces and suspended with appropriate amount of distilled water and then homogenized by using a basic homogenizer. The homogenate was heated at 90°C for 15 for 15 min. After heating the tissue homogenate hydrolyzed with protease and Flavourzyme followed by cultivating with dry yeast at 35°C for 60 min and heat inactivating at 95°C for 10 min. the supernatant was collected by centrifugation and lyophilized to obtain the powder form. The total nitrogen and polypeptide contents and molecular mass of SPP was determined using gel filtration chromatography as describe by Jiang TC et.al (Jiang et al., 2014).

### Animals

Male Kunming mice weighting 21–27 g were housed under standard laboratory conditions, provided with standard diet and drinking water ad *libitum*. Animal experiments were approved by TUTCM Ethical Review Committee and carried out in accordance with international guidelines for care and use of laboratory animals. After 1 week of acclimatization the mice were randomly divide in to five groups, 10 mice in each group as follow; normal control (mice without drug and without forced swimming), model (mice without drug but with forced swimming), SPP-200 (mice taking 200 mg/kg SPP and with forced swimming), SPP-400 (mice taking 400 mg/kg SPP and with forced swimming), positive control (mice taking 200 mg/kg CoQ10 and with forced swimming). SPP was dissolved with 0.5 ml of normal saline, orally given via gavage daily for 1 month, normal control and model groups were only administered normal saline as a vehicle. Swim screening were undergone two times to eliminate the mice which unable to swim.

### Forced Swimming Test

After administration of the drug for 1 month and after 30 min of the last intragastric administration, 10 mice were taken from each group and placed individually in to a plastic swimming pool containing 30–40 cm deep water with the temperature maintained at 30 ± 2°C. The mice were forced to swim till exhaustion for 90 min. After 1 h rest all animal were anesthetized with 10% chloral hydrate and blood were collected from eye ball. Serum was extracted and stored at -80°C and red gastrocnemius muscle was collected from the hind limbs. Aliquots of each sample were quickly snap freezed in liquid nitrogen and stored at -80°C for further analysis until required.

### Electron Microscopic Examination of Mitochondrial Structure

Small pieces of fresh skeletal muscle (∼1 mm) from mice hind limb were fixed with 6% glutaraldehyde, 6% valeric aldehyde fixatives then post fixed with 1% osmium tetroxide, dehydrated, and embedded in EPON. Ultrathin (500 nm) longitudinal and transverse sections were cut for each sample using ultra-thin slicing machine (LEICA-EM UC7, Germany). The sections stained with lead citrate and uranyl acetate. Longitudinal and transverse sections of the specimen were examined using transmission electron microscopy (H600 Hitachi). Microphotographs were randomly taken from each block then mitochondrial morphology and structure were analyzed.

### Tissue MDA and Mitochondrial SOD (MnSOD) determination

Mice skeletal muscle MDA was determined to quantify lipid peroxidation by measuring reaction of tissue MDA with TBA to form blue colored formazan in presence of phenazine metha sulphate (PMS) and NADH using microplate reader (thermo scientific Varioskan).

For determination of mitochondrial SOD level, mitochondria were isolated from muscle homogenates by differential centrifugation based on manufacturer kits protocol. Briefly skeletal muscle from mice hind limb were excised and homogenized with appropriate medium. The homogenates were centrifuged at 600 g for 5 min to remove cell debris and the nuclear fraction. And the resultant supernatant was centrifuged at 10,000 g for 10 min to sediment mitochondria. Mitochondrial sediment was ultra-sonicated (at 20% power, 3 s, Interval of 10 s, 30 times). Protein was estimated and pellets were suspended in appropriate buffer solution. Measurement of mice mitochondrial SOD level was estimated by following reduction of nitroblue tetrazolium (NBT) using microplate reader.

### Inflammatory Cytokines Level Determination

A Quantitative analysis of serum TNF-α and IL-6 level were measured based on standard sandwich enzyme-linked immune-sorbent assay technology. The absorbance of yellow color was detected at 450 nm using microplate reader.

### Cell Viability Assay

Over all cell viability of SPP was measured by MTT assay as follow; L6 cell were seeded into 96 well culture plates at a density of 3 × 10^4^ cells per well and in α-MEM medium supplemented with 10% FBS 1% NEAA and 1% P/S, incubated for 24 h. After adherence for 24 h, the cell were exposed to different concentrations of SPP (50, 100, 500 ng/ml, 1, 2, 4, 6, 8, 10, 15, 25, 50, 75, 100, 200, 400, 600, 800 μg/ml, 1 mg/ml) and incubated for additional 24 h. After incubation, the assay media was replaced with 10 ul of MTT solution. 5 mg/ml final concentration of MTT was added to each well and the cells were allowed to incubate at 37°C for an additional 4 h. The media was removed, and the resulting formazan crystals were solubilized in in 100 ul DMSO. The absorbance was measured at 570 nm using microplate reader. The number of living cells in the treated groups was expressed as a percentage of the control group; (absorbance of drug/absorbance of control) × 100. All assays were performed with triplicates.

### Statistical Analysis

All experimental data were expressed as mean ± SEM and statistically assessed by one-way analysis of variance (ANOVA). The differences between measurements were analyzed using student *t*-test. *P*-value < 0.01 was considered to be statistically significant. SPSS software (version 13.0.1; SPSS Inc, Chicago, IL,USA) and Graph Pad (GraphPad Prism 5.lnk version 5.03) were used for all statistical analyses.

## Result

### SPP Maintain Mitochondrial Dynamic Network

**FIGURE 1:**
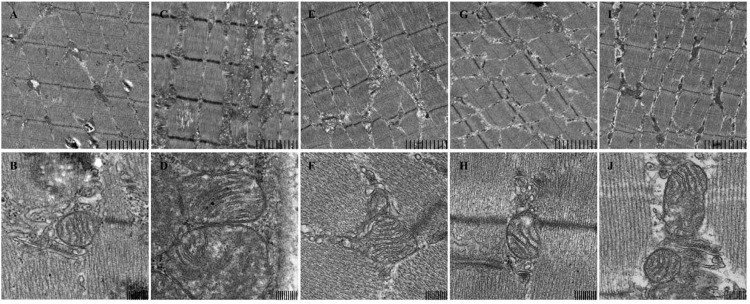
**Mitochondrial structure of mice skeletal muscle under electron microscopy. (A,B)** control, **(C,D)** model, **(E,F)** SPP-200 mg/kg, **(G,H)** SPP-400 mg/kg, **(I,J)** CoQ 10 200 mg/kg at 4.0 K (upper) and 20.0 K (lower) magnification. Scale bar represent 2 um and 200 nm respectively.

Ultra structural analysis of mice skeletal muscle biopsies revealed an enormous change in mice skeletal muscle mitochondrial structure in model group compared to normal control group. Generally Model group showed various abnormalities including increased number and size of mitochondria, pleomorphism, compartmentalization, and zigzag cristae as shown in (**Figure 1**). This proves use of forced swimming model to induce mitochondrial dysfunction due to chronic fatigue worked very well. Whereas, in SPP 200 mg/kg and 400 mg/kg the size of mitochondria is almost similar to control group while in positive control (CoQ10) group mitochondrial size is slightly larger than normal control group but smaller compared to model group. The other most remarkable abnormality seen was on internal structure of mitochondria which produced an appearance of compartmentalization, apparently produced by branching and fusion of the cristae. In model group almost all mitochondrion’s lost their normal parallel arrangement of cristae at the sites of the branching and fusion, some of mitochondrion’s in SPP 400 mg/kg and positive control (CoQ10) group also lost some of their parallel arrangement of cristae, only occasional mitochondria showed compartmentalization. But in SPP 200 mg/kg the arrangement of cristae was as intact as normal control group. Moreover the outer and inner membrane of mitochondria was lysed in forced swimming model group while it was intact in rest of other group. The results have shown SPP have protected mitochondrial dysfunction by maintaining the normal structure and through preserving outer and inner membrane integrity.

### Effect of SPP on ROS

#### SPP Inhibited Lipid Peroxidation

**FIGURE 2:**
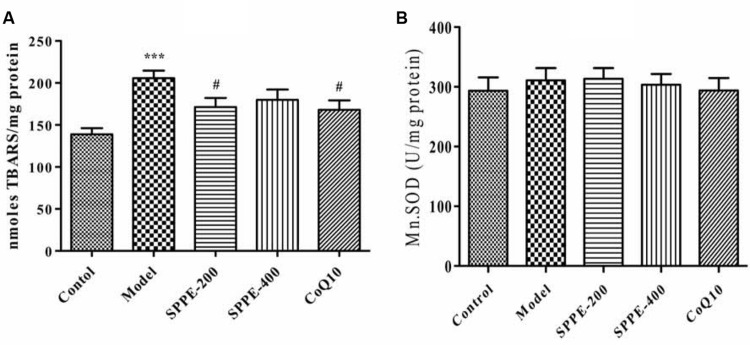
**(A)** Effect of SPP on mice skeletal muscle MDA level. **(B)** Effect of SPPE on mice skeletal muscle mitochondrial SOD level each bar represent Mean ± SEM (*n*=10). Significant at ***p-value < 0.001 compared to control; #*p*-value < 0.05 compared to model.

Forced swimming for 90 min resulted in a significant increase in lipid peroxidation; as measured by the formation of TBARS (**Figure 2** and **Table 1**). There is a highly significant increase of tissue MDA level (*P* < 0.001) in model group (205.68 nmol/mg protein) which undergo forced swimming compare to normal control group (138.84 nmol/mg protein). The current results also demonstrated that SPP inhibited lipid peroxidation at both high and low concentration 171.38 nmol/mg protein and 179.89 nmol/mg protein, respectively (**Figure 2** and **Table 1**) SPP at 200 mg/kg significantly reduce TBARAS level (*P* < 0.05) but SPP at 400 mg/kg was not statistically significant. Positive control group (CoQ10 200 mg/kg) has also significantly inhibited lipid peroxidation (168.07 nmol/mg protein) compared to model group (*P* < 0.05).

**Table 1:** MnSOD and MDA level of mice skeletal muscle.

	MDA (nmoles TBARS/mg protein)	Mn.SOD level (U/mg protein)
Control	138.84 7.26	293.45 22.45
Model	205.68 8.93ˆ***	310.70 20.81
SPP-200	171.38 10.76#	313.42 18.00
SPP-400	179.89 12.30	303.57 17.88
CoQ10	168.07 11.26#	293.87 21.00

Results are expressed as mean ± SEM (n = 10). Significant at ***p-value < 0.001 compared to control; #p-value < 0.05 compared to model.

#### SPP Has No Effect on Mitochondrial SOD Level

Measurement of MnSOD was done to examine the effect SPP on mitochondrial anti-oxidant level. As seen from the result (**Table 1** and **Figure 2**) forced swimming doesn’t induced any significant change compared to normal control mice, as well as there is no significance difference between all treatment groups.

### Effect of SPP in Inflammatory Cytokines Level

#### SPP Increase Serum TNF-α Level

**FIGURE 3:**
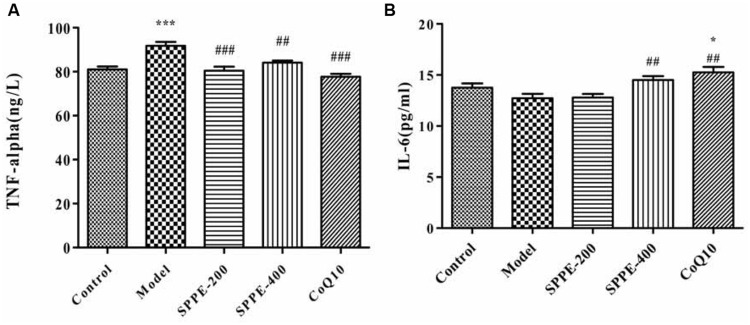
**(A)** Effect of SPP on mice serum TNF-α level. **(B)** Effect of SPP on mice serum IL-6 level. Each bar represent M±SEM (*n*=10). Significant at ***p-value < 0.001 compared control; ###*p*-value < 0.001, ##*p*-value < 0.01 compared to model. * indicates significance compared to control.

To elucidate whether forced swimming could increase serum TNF-α level, and to see the effect SPP on cytokines level serum TNF-α level was measured as shown in (**Figure 3** and **Table 2**). Forced swimming of mice for 90 min resulted in a significant increase in serum TNF-α level of model group (91.82 ng/l) compared to normal control group (81.07 ng/l) *P*-value < 0.001. On the other hand, treatment of mice with SPP 200 and 400 mg/kg significantly reduced serum TNF-α level 80.42 and 84.12 ng/l, respectively. SPP at lower dose inhibited serum TNF-α level (*P* < 0.001) better than SPP at high dose (*P* < 0.01) compared to normal control group. Moreover, positive control mice taking CoQ10 200 mg/kg have also showed a significant inhibition of TNF-α (*P*-value < 0.001).

**Table 2:** Mice serum TNF-α and IL-6 level.

	TNF-α(ng/L)	Serum IL-6(pg/ml)
Control	81.07 1.28	13.75 0.42
Model	91.82 1.72ˆ***	12.72 0.43
SPP-200	80.42 1.85###	12.79 0.35
SPP-400	84.12 0.96##	14.51 0.35##
CoQ10	77.70 1.33###	15.25 0.55##

Results are expressed as mean ± SEM (n = 10). Significant at ***p-value < 0.001 compared control; ###p-value < 0.001, ##p-value < 0.01 compared to model.

#### SPP Increase Serum IL-6 Level

Forced swimming decreased serum IL-6 level (12.72 pg/ml) compared to normal control group (13.75 pg/ml) but the difference doesn’t display any statistical significance (**Figure 3** and **Table 2**). However administration of SPP at 400 mg/kg significantly increased serum IL-6 level (14.51 pg/ml) compared to model group (*P*-value < 0.05) whereas there was no significant difference compared to normal control group. Moreover mice which take 200 mg/kg CoQ10 have also showed a significant increase in serum IL-6 level (15.25 pg/ml) compared to both model group (*P*-value < 0.01) and normal control group (*P*-value < 0.05). Although SPP at lower dose (200 mg/kg) increase serum IL-6 level (12.79) compared to model group, it did not show any significant difference with both control and model group.

#### Effect of SPP on Cell Viability

**FIGURE 4:**
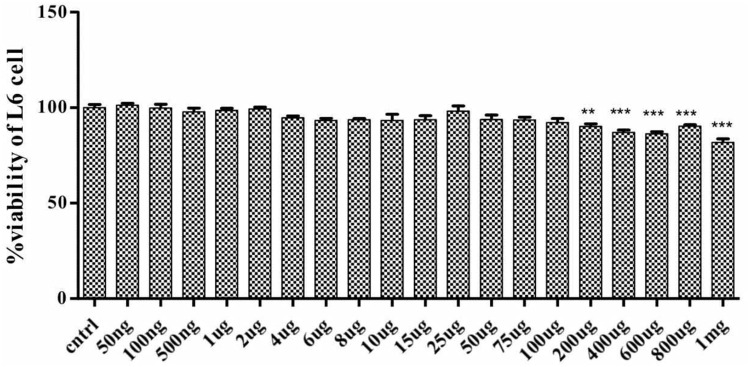
**Effect of SPP (200 ng/ml-l mg/ml) in L6 cell viability.** Each bar represents percentage of cell viability. SPP 200 ng/ml-l mg/ml has significantly reduced the cell viability. Significant at ***p-value < 0.001 and **p-value < 0.01.

To determine the effect of SPP on cell viability and cell death, rat skeletal muscle L6 cell were treated with different concentration of SPP ranging from 1 mg/ml to 50 ng/ml for 24 h. SPP didn’t show any significant change in the cell viability of L6 cell ranging from 100 μg/ml to 50 ng/ml (**Figure 4**). However, it promoted cell death in dose-dependent manner, at 200 μg/ml (*P*-value < 0.01) and above (*p*-value < 0.001).

## Discussion

Several studies have revealed that placenta extract can inhibit fatigue, aging and many other diseases (Biswas et al., 2001; Kong et al., 2008; Kong and Park, 2012; Rozanova et al., 2012). Placenta was also reported to have a possible anti-oxidant and immune-modulating effect in various biological systems (Park et al., 2010; Kawakatsu et al., 2013). Peptides as well have anti-oxidative components, can hinder fatigue and protect mitochondrial viability and minimize further ROS generation (Togashi et al., 2002; Wang et al., 2008; Ren et al., 2011; You et al., 2011).This properties of placenta and peptide may play an important role on mitochondrial functioning. Therefore this study examines the effect of SPP on mitochondria during chronic fatigue induced by forced swimming.

Mitochondria are the primary controllers of cellular metabolism and form a reticular network within mammalian skeletal muscle (Brookes et al., 2004; Pieczenik and Neustadt, 2007; Neustadt and Pieczenik, 2008). Hence defect in mitochondrial membrane and the general structure will cause mitochondrial dysfunction resulting fatigue seen in chronic fatigue patients (Russell et al., 2014). The current finding also elucidated forced swimming can cause a massive damage in both inner and outer mitochondrial membranes of mice skeletal muscle triggering release of mitochondrial components that will result necrosis and cell death. However, pretreatment with SPP prevented skeletal muscle mitochondrial structure from damage; SPP shown to protect mitochondrial dynamic network by maintaining inner and outer membrane integrity.

Moreover, there is an abundant evidence that elevated production of ROS increases in exceedingly contracting skeletal muscle during strenuous exercise due to stress (Malm, 2002; Urso and Clarkson, 2003; Jammes et al., 2009; Finsterer, 2012). In addition, animal studies provide convincing evidence that ROS contribute to muscle fatigue induced by prolonged muscular contractions (Park et al., 2010; Wang et al., 2010). In this study, a highly significant increase in skeletal muscle MDA level was found in fatigue model of forced swimming compare to normal control group. This elevated level of MDA indicates that there is a high lipid peroxidation in the cell membrane due to attack of free radicals. Besides, mitochondrial dysfunction is one of the main causes of oxidative stress mainly induce as a result of elevated level of ROS, and the main source of ROS generation is the mitochondria (Balaban et al., 2005; Pieczenik and Neustadt, 2007; Norheim et al., 2011; Indo et al., 2015). For this reason forced swimming cause lipid peroxidation by elevating generation of ROS, that will attack mitochondrial components resulting damage which in turn will cause more elevated level of ROS, that will make the mitochondrial dysfunction worse. In contrast pretreatment of experimental animal with SPP inhibited increment of skeletal muscle MDA level. Since lesser level of tissue MDA indicate low lipid peroxidation and low ROS generation, SPP has inhibited lipid peroxidation which will also cause lower level of ROS and protect damage to mitochondrial component.

Fatigue is also related to production of inflammatory cytokines, immune dysfunction, and cytokine imbalances (Northoff and Berg, 1991; Cannon and St Pierre, 1998; Suzuki et al., 2002; Doong et al., 2014; Nijs et al., 2014; Peterson et al., 2015). Several studies have demonstrated muscle tissue damage after sternness physical exercise, as well as muscle damage regardless of the cause will induce an inflammatory response (Tidball, 1995; Huard et al., 2002; Sugama et al., 2015). Moreover administration of cytokines to smooth muscle cells reportedly inhibits mitochondrial respiration causing mitochondrial dysfunction (Chazotte, 2001). This evidence suggested that cytokines may affect mitochondrial function during chronic fatigue. Our finding also supports all the above idea; forced swimming result a dramatic elevation of serum TNF-α level. Since mitochondria is the main source of ROS generation required for TNF-α induced cell death, an abnormal level of TNF-α has a profound effect on mitochondria causing a marked and prolonged decrease, on mitochondrial and cellular bioenergetics (Kim et al., 2010; Babu et al., 2015). TNF-α generates ROS at the mitochondrial inner membrane, which may easily result in the progressive destruction of the mtDNA, as mtDNA lacks the structural protection of histones and their repair mechanisms; therefore, they are quite susceptible to damage due to high level ROS generated by TNF-α resulting mitochondrial dysfunction during chronic fatigue (Corda et al., 2001; Suematsu, 2003; Kim et al., 2010). As discussed in the above literatures, elevation of TNF-α cause generation of ROS that will attack mitochondrial components resultng mitochondrial dysfunction, as well as chronic fatigue. Hence, forced swimming model has profoundly induced mitochondrial dysfunction and chronic fatigue through triggering elevated level of TNF-α secretion, whereas, SPP significantly attenuated serum TNF-α secretion in mice during chronic fatigue. Reduction of TNF-α due to SPP treatment may also result reduction of ROS generation in mitochondria, this will allow proper functioning of mitochondria, that will in turns inhibit progression of fatigue seen in chronic fatigue.

On the other hand, extreme physical stress displayed promotes the systemic release of cytokine antagonists and factors that inhibit cytokine synthesis in order to prevent disturbance of homeostasis due to systemic inflammation (Suzuki et al., 2002). Researchers have showed that serum IL-6 level can be increased during extreme physical stress (Malm, 2002; Finsterer, 2012; Azizbeigi et al., 2015), but unlike TNF-α its elevation do not to raise ROS level and not related to muscle damage, rather IL-6 have a function linked to energy metabolism by activation of glycogenolysis, it displayed to increase glucose uptake during stress (Malm, 2002; Ando et al., 2010; Finsterer, 2012). Moreover, IL-6 has a major role as an anti-inflammatory cytokines; it has a down-regulatory effect on TNF-α and other proinflammatory cytokines production. It can block the synthesis and action of TNF-α (Tackey et al., 2004; Brandt and Pedersen, 2010). However, IL-6 level did not increase in chronic fatigue patients during excessive exercise (Jammes et al., 2009). This finding also agreed with the above literatures, there is no significant change in serum IL-6 level in forced swimming fatigue animal model. but treatment of experimental animal with SPP can significantly increase serum IL-6 level, that may linked to its effect to inhibit mitochondrial ROS production through suppressing TNF-α elevation during oxidative stress. In addition exhaustion theory suggests that during stressed exercise, many energy sources, such as glucose and liver glycogen, will be exhausted, thus leading to physical fatigue (Wang et al., 2008). Several reports showed that through the administration of proteins or peptides can facilitate recovery from fatigue (Wang et al., 2006; You et al., 2011).Thus, increment of IL-6 level with SPP may increase glucose up take to alleviate stresses that occur during chronic fatigue.

## Conclusion

**FIGURE 5:**
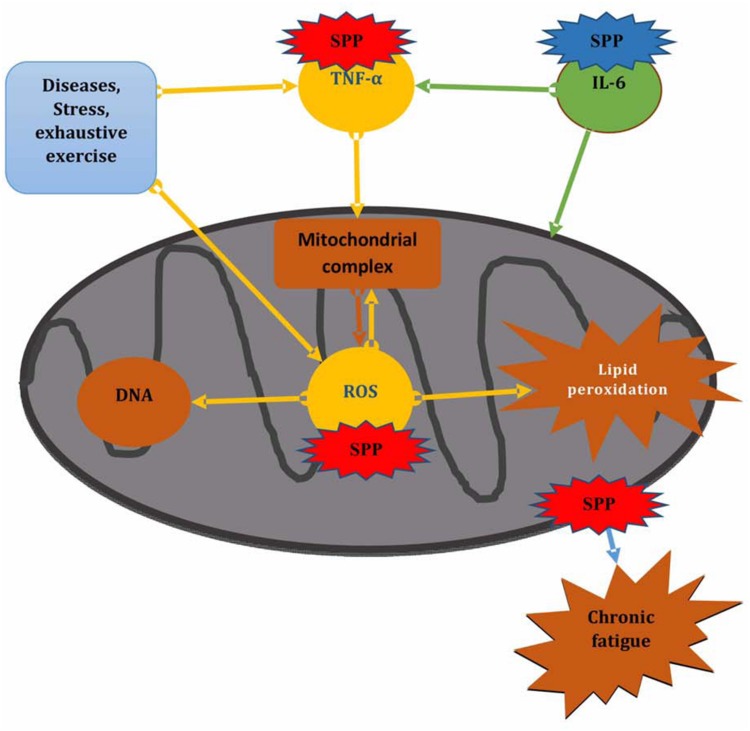
**Effect of SPP on mitochondrial functioning during chronic fatigue.** SPP in red color shows inhibitory effect, SPP in blue color shows promotor effect.

Generally our finding revealed that forced swimming chronic fatigue model can cause mitochondrial dysfunction by increasing TNF-α level as well as attacking mitochondrial component through ROS induced lipid peroxidation and also by disturbing mitochondrial dynamic network. Whereas, SPP showed to protect forced swimming induced mitochondrial dysfunction and protect damage to mitochondrial component through preventing ROS and TNF-α generation, by maintaining mitochondrial dynamic network and by increasing serum IL-6 level, that may linked to its effect to inhibit mitochondrial ROS and also increase glucose up take to alleviate stress that occur during chronic fatigue (**Figure 5**).

All of these findings support mitochondria as new pharmacological targets for treating chronic fatigue and SPP may represent a novel therapeutic advantage for preventing mitochondrial dysfunction in patients with chronic fatigue.

## Author Contributions

Title selection, proposal writing and research design: RM and YB Laboratory experimentation, result generation, data analysis, and interpretation: RM, YB, LW, PA, HC, XP, and SL. Manuscript writing and submission: RM Advising and edition in proposal and research design, result interpretation, and manuscript writing: YB.

## Conflict of Interest Statement

The authors declare that the research was conducted in the absence of any commercial or financial relationships that could be construed as a potential conflict of interest.

## Abbreviations

CF: Chronic fatigue
CoQ10: Coenzyme Q10
ETC: Electron transport chain
FADH: Flavin adenine dinucleotide
H2O2: Hydrogen peroxide
HO^-^: Hydroxyl radical
MDA: Malondialdehyde
MDA: Malondialdehyde
MnSOD: Manganese Superoxide dismutase
MTT: Thiazoyl blue tetrazolium
NADH: Nicotinamide-adeninedinucleotide
NADH: Nicotinamide-adeninedinucleotide
ROS: Reactive oxygen species
SPP: Sheep placenta peptide
TBA: Thiobarbituric acid
TNFαa: Tumor Necrosis factor alpha
TUTCM: Tianjin University of Traditional Chinese Medicine.
